# Editorial: Prehospital emergency medicine: challenges and opportunities

**DOI:** 10.3389/fmed.2024.1518523

**Published:** 2024-11-14

**Authors:** Sebastian Schnaubelt, Enrico Baldi, Mario Krammel, Patrick Sulzgruber

**Affiliations:** ^1^Emergency Medical Service, Vienna, Austria; ^2^Department of Emergency Medicine, Medical University of Vienna, Vienna, Austria; ^3^PULS – Austrian Cardiac Arrest Awareness Association, Vienna, Austria; ^4^Division of Cardiology, Fondazione IRCCS Policlinico San Matteo, Pavia, Italy; ^5^Cardiac Arrest and Resuscitation Science Research Team, Fondazione IRCCS Policlinico San Matteo, Pavia, Italy; ^6^Division of Cardiology, Department of Internal Medicine II, Medical University of Vienna, Vienna, Austria

**Keywords:** emergency medicine, emergency medical service (EMS), ambulance, prehospital/EMS, emergency care

Emergency medical care provided before hospital arrival depends on functioning emergency medical services (EMS) and covers a wide range of medical and organizational topics (1). No matter whether a system is physician-, nurse-, or purely paramedic-based or whether it more follows an Anglo-American or Franco-German style (2), the ultimate goal of a system that saves lives is to strive for excellency. A skill transfer including (non-)invasive techniques and novel technologies from the in- to the pre-hospital setting has in recent years been increasingly shown to improve the quality of care and, subsequently, outcomes. Successful examples include blood gas analysis, point-of-care ultrasound, and video laryngoscopy (3–5). To further advance these endeavors, research is not only a factor of pre-hospital medicine that is “nice to have” but a definitive necessity (1, 6). Concepts such as research units have in the past been shown to be productive solutions, but research can also be integrated into the everyday EMS work routine. However, several challenges have emerged in this context, for instance adapting clinical research methodology to the pre-hospital setting. Limited resources and EMS organizations not being a traditional part of academia pose further barriers (6, 7).

The Research Topic we hereby present aims at showcasing the progress made across the wide variability of prehospital emergency medicine.

## Education

Armijo-Rivera et al. present a study on the Team Emergency Assessment Measurement (TEAM) and its novel Spanish version's applicability in Chile. TEAM assesses non-technical performance as a self-reporting tool. This was found to be feasible and highlights the possibility to apply scoring systems in different populations. Izquierdo-Condoy et al. took one step further and assessed the preparedness and proficiency of life support. Included nursing staff showed a relevant deficiency in theoretical basic and advanced life support knowledge. Even though performed with nursing staff, the assessment process via questionnaires is well-described and could also be conducted with pre-hospital personnel.

## Trauma

Teuben et al. assess the impact of pre-hospital trauma life support (PHTLS^®^) course participation on altered self-confidence, communication, and routines in the treatment of severe trauma and included a mix of paramedics and emergency physicians. PHTLS^®^ training was associated with improved self-confidence and enhanced communication, implying that pre-hospital personnel, and ultimately also patients, profit from standardized course involvement. Lin et al., on the other hand, investigate the more specific pathology of traumatic lung injury (TLI). In more than 100 TLI patients, the international standardized ratio (INR) was identified as a marker for poor prognosis and was suggested as an early warning indicator—a factor that could be taken into account in future thoughts about point-of-care testing in the pre-hospital setting.

## Cardiopulmonary resuscitation

Skrisovska et al. conduct a simulation study to assess the efficacy of ventilation during pediatric cardiopulmonary resuscitation (CPR). Both healthcare professionals (using bag-valve-mask ventilation) and lay rescuers (using mouth-to-mouth ventilation) were included. Assisted by dispatchers, participants could deliver effective ventilation to pediatric manikins, underscoring the feasibility of current respective guidelines.

## Ethics

Lemoyne et al. tackle a part of pre-hospital emergency medicine that has always been and increasingly will be of the utmost importance: nursing homes and their hospitalization approaches, in which a proportion of nursing home interventions and hospital transfers might be preventable. While this topic can be approached from an economic perspective, the EMS personnel's perceptions of the adequacy of emergency calls from nursing homes is potentially even more relevant. EMS staff reported that nursing home interventions were rarely necessary or indicated, and various key issues and potential solving strategies were identified.

## Forecasting emergencies

Yang et al. took an unusual look at a part of pre-hospital care that is often underrepresented in literature: maritime emergency medicine. Due to the extremely limited resources in this setting, emergency forecasting models could potentially help organize underlying systems. Managed maritime emergency cases were analyzed, and various models were assessed to forecast emergencies. According to the authors, this could be a steppingstone in facilitating the development of effective prevention and control strategies.

To conclude, it is of the utmost importance to further promote pre-hospital research in various resource settings and environments. Especially against a background of emerging pre-hospital care in low-resource settings and the adjacent challenges (8, 9), novel strategies of evidence gathering, evidence synthesis, and the implementation of evidence-based approaches in pre-hospital emergency medicine will remain a “hot topic” for quite some time.
